# Correction: *Anoplophora graafi* longhorn beetle coloration is due to disordered diamond-like packed spheres

**DOI:** 10.1039/d5sm90030a

**Published:** 2025-02-18

**Authors:** Kenza Djeghdi, Cédric Schumacher, Viola Bauernfeind, Ilja Gunkel, Bodo D. Wilts, Ullrich Steiner

**Affiliations:** a Adolphe Merkle Institute, University of Fribourg Chemin des Verdiers 4 1700 Fribourg Switzerland ullrich.steiner@unifr.ch; b National Competence Center in Bioinspired Materials, University of Fribourg Chemin des Verdiers 4 1700 Fribourg Switzerland; c Department of Chemistry and Physics of Materials, University of Salzburg Jakob-Haringer-Straße 2A 5020 Salzburg Austria bodo.wilts@plus.ac.at

## Abstract

Correction for ‘*Anoplophora graafi* longhorn beetle coloration is due to disordered diamond-like packed spheres’ by Kenza Djeghdi *et al.*, *Soft Matter*, 2024, **20**, 2509–2517, https://doi.org/10.1039/D4SM00068D.

The authors regret the omission of an acknowledgement in the original article. This acknowledgement is given below.

We acknowledge the European Synchrotron Radiation Facility for provision of synchrotron radiation facilities, and we would like to thank Dr Lauren Matthews for assistance in using beamline ID-02 (under proposal SC-5238).

The Royal Society of Chemistry apologises for these errors and any consequent inconvenience to authors and readers.

