# Supplementary material for: Anoplophora graafi longhorn beetle coloration is due to disordered diamond-like packed spheres
Source: Soft Matter. 2024 Feb 19;20(11):2509–17. doi: 10.1039/d4sm00068d (PMC10933740; doi:10.1039/d4sm00068d)
Supplement: SM-020-D4SM00068D-s001 [file SM-020-D4SM00068D-s001.pdf]

This journal is © The Royal Society of Chemistry 2024

Supplementary Information

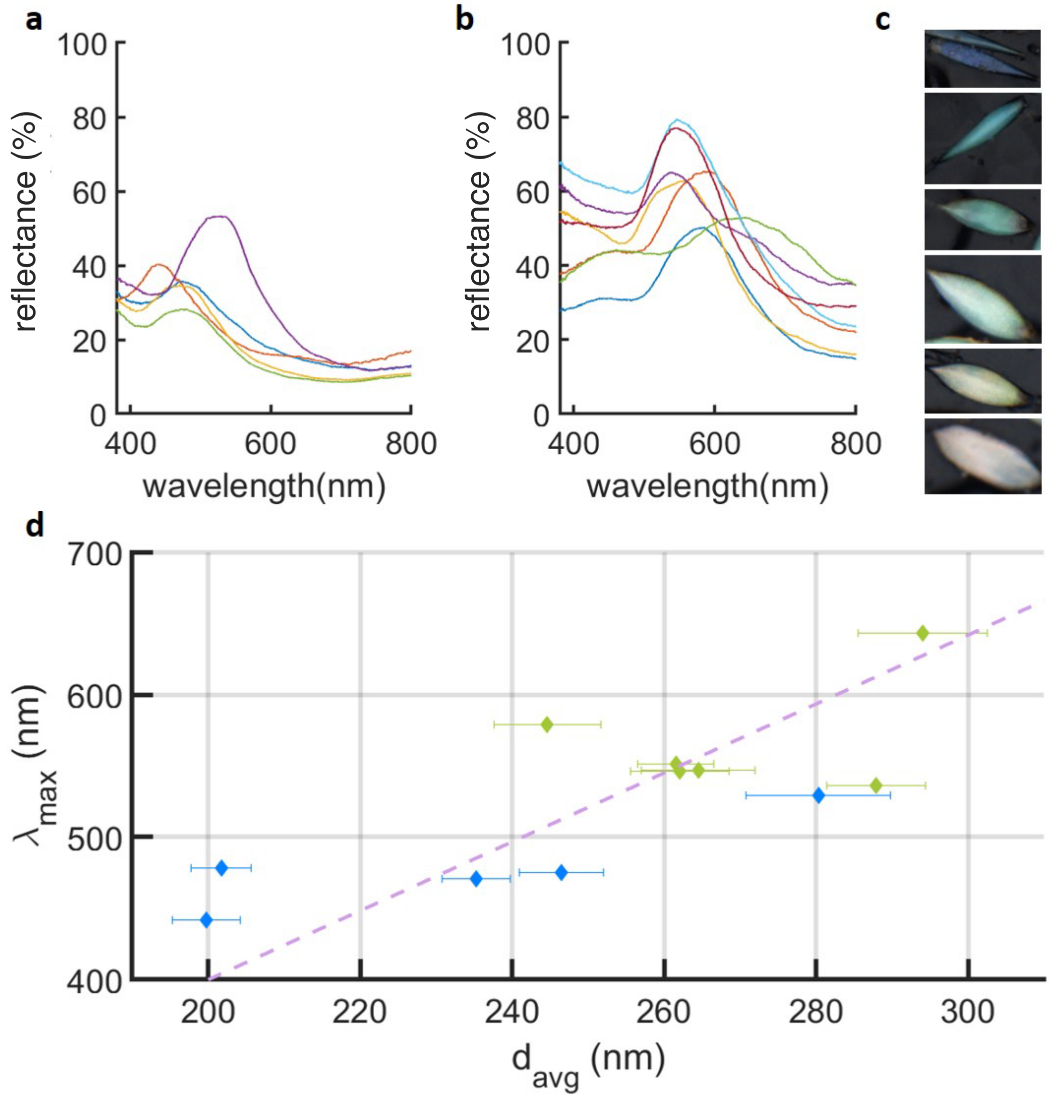

Fig. S1 Relationship between sphere diameter and reflected wavelength. (a) Reflectance spectra of scales collected from legs and (b) the main body, measured against a white diffusive reference tile. (c) Examples of scales covering the entire visible wavelength range. (d) Plot of the peak reflectance wavelength λ_{max} vs. the average sphere diameter d_{avg}. The sphere diameter is an average from SEM image analysis using Fiji (N=15). The regression line is a guide to the eye.

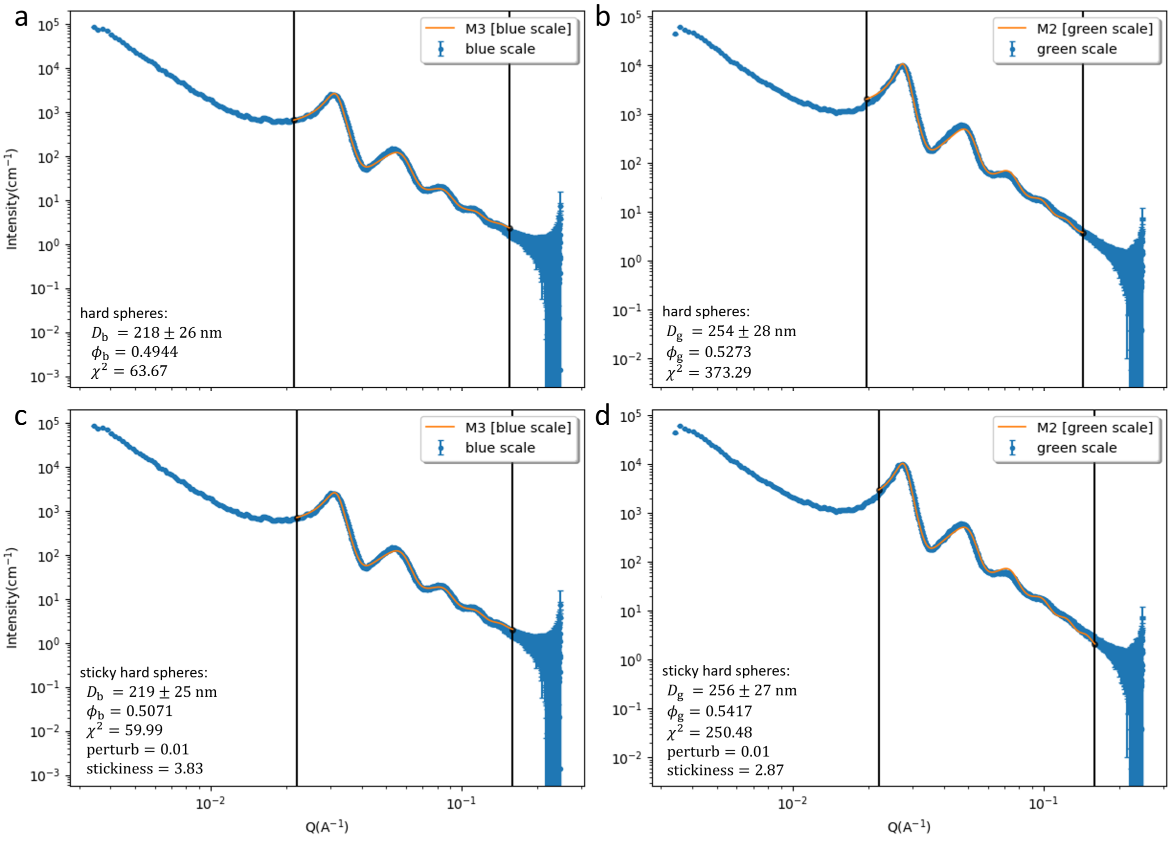

Fig. S2 SAXS intensity profiles of (a,c) green scales and (b,d) blue scales with two different models. The fitted hard sphere (a,b) and sticky sphere (c,d) models, represented by the straight lines, fit the data well, and the corresponding fit parameters are detailed in the insets. Both models yield nearly identical sphere diameters, with minor variations in volume fractions and a slightly improved fit for the sticky hard sphere model.

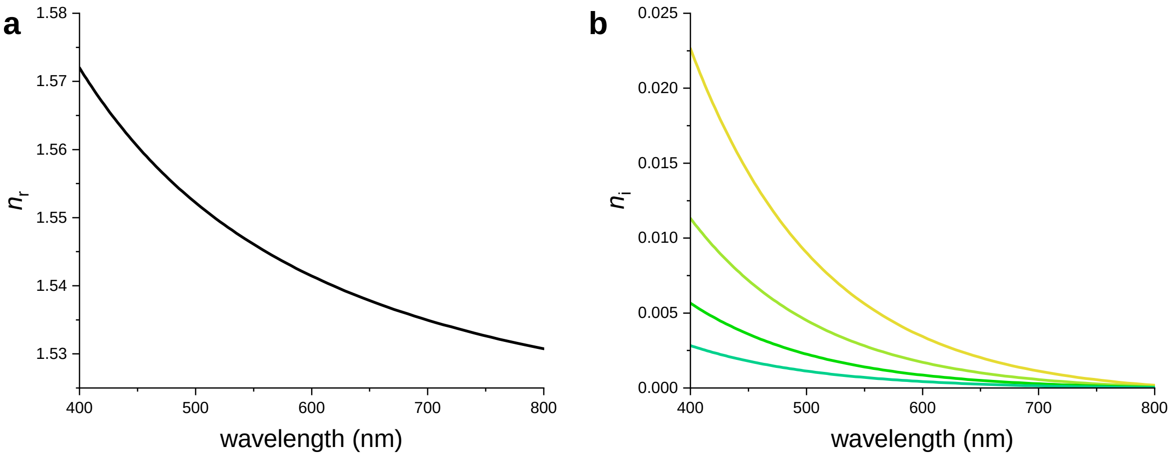

Fig. S3 Refractive index dispersion as used in the FDTD modelling of Fig. 4. Due to the relatively small amount of pigment, the real part of the refractive index was assumed to be identical to cuticular chitin[22] and the imaginary part was varied. Color scheme of the curves is identical as in Fig. 4.

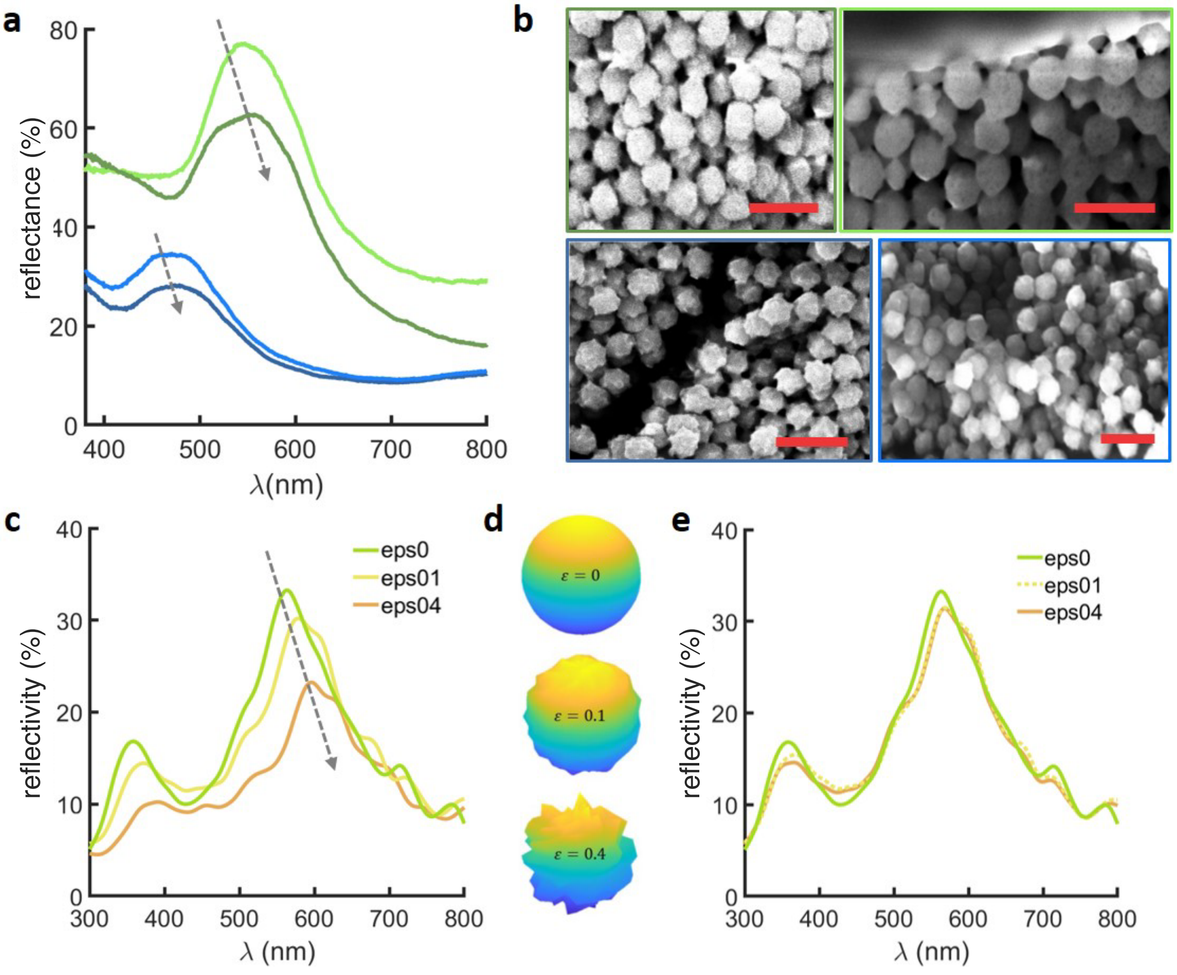

Fig. S4 Effect of sphere roughness. (a) Reflectance spectra from two green and two blue scales, shown in (b), containing spheres with differing surface roughness. The spectra of the rougher spheres are slightly red-shifted with a lower overall reflectance (green scale: $\Delta\lambda = 14\,\text{nm}$, $\Delta R = 14\%$; blue scale: $\Delta\lambda = 4\,\text{nm}$, $\Delta R = 7\%$). The scale bars in (b) are 500 nm. (c) FDTD simulated reflectivity spectra for the tomogram of Fig. 2b,c, replacing the measured objects with perfectly smooth spheres and adding increasing surface roughness, as indicated in (d). The increasing spectral red-shift with increasing surface roughness arises from the corresponding decrease in sphere volume. When scaling the spheres to the same volumes in (e), keeping the overall filling volume fraction equal, the spectra of smooth and rough spheres overlap.
